# WebCM: A Web-Based Platform for Multiuser Individual-Based Modeling of Multicellular Microbial Populations and Communities

**DOI:** 10.1021/acssynbio.3c00486

**Published:** 2024-05-14

**Authors:** Jason Philippou, Guillermo Yáñez Feliú, Timothy J. Rudge

**Affiliations:** Interdisciplinary Computing and Complex Biosystems, School of Computing, Newcastle University, Newcastle upon Tyne NE1 7RU, U.K.

## Abstract

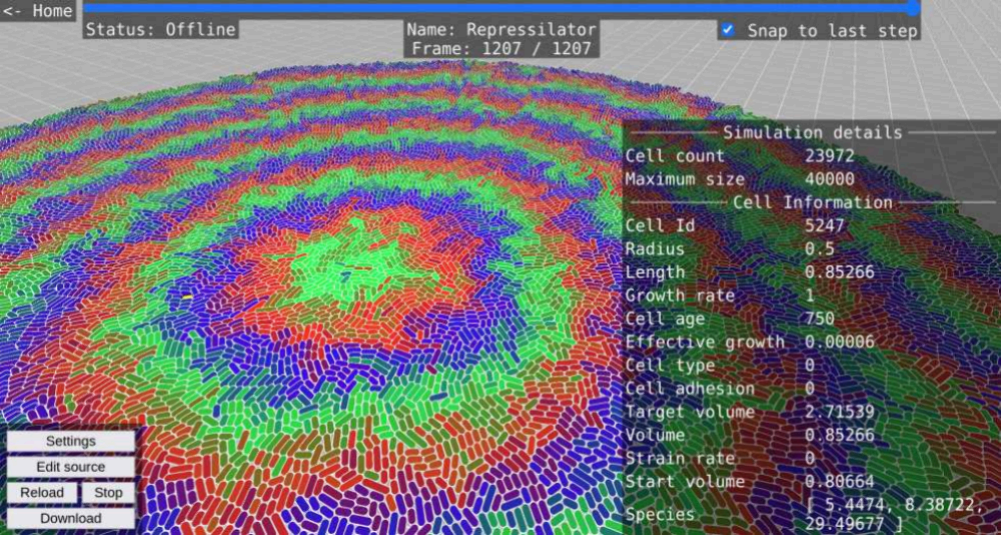

WebCM is a web platform that enables users to create, edit, run, and view individual-based simulations of multicellular microbial populations and communities on a remote compute server. WebCM builds upon the simulation software CellModeller in the back end and provides users with a web-browser-based modeling interface including model editing, execution, and playback. Multiple users can run and manage multiple simulations simultaneously, sharing the host hardware. Since it is based on CellModeller, it can utilize both GPU and CPU parallelization. The user interface provides real-time interactive 3D graphical representations for inspection of simulations at all time points, and the results can be downloaded for detailed offline analysis. It can be run on cloud computing services or on a local server, allowing collaboration within and between laboratories.

## Introduction

Cellular-scale individual-based models (IBMs)^1^ give rich information about the emergent collective properties of cell populations due to the interactions between themselves and their environment. These models track cell growth and gene expression in ways analogous to experiments performed under controlled environmental conditions with specified properties such as their geometry, viscosity, chemical concentrations, etc. This makes them an essential tool in the analysis and design of emergent properties of synthetic and natural genetic circuits operating in a multicellular chassis. However, their accessibility to nonprogrammers is limited, and they may require specialist hardware to run sufficiently large simulations.

CellModeller^2^ is a Python framework for simulating such IBMs that requires minimal Python scripting abilities to construct and run models. It has been applied to model and analyze many multicellular behaviors of microbial populations, including the effect of contact-dependent inhibition (CDI) systems at the single-cell level,^3^ emergent properties due to physical interactions between cells,^4^ interactions between antibiotic-resistant and susceptible strains in the pathogen *Pseudomonas aeruginosa*,^5^ impact of cell morphology on microbial community structure,^6^ influence of adhesive intercellular interactions on biofilm spatial structure,^7^ understanding the evolutionary function of bacterial weapons and their effect on the function of probiotics,^8^ symmetry-breaking and domain-specific cell regulation as elementary functions for the prototyping of morphogenetic programs,^9^ formation of traveling waves of gene expression due to growth rate heterogeneities in colonies,^10^ and training deep convolutional neural networks (CNNs) using simulated biofilm images for automated morphometric cell classifications in multipopulation biofilms.^11^

In order to run large simulations required to model typical bacterial populations, CellModeller requires a processor with multiple cores or a graphics processing unit (GPU) enabling parallelization of the core algorithms. This limits accessibility for typical users. This may be overcome to some extent by running simulations on a server or workstation by a remote connection (remote desktop or command line). However, this does not allow users to easily inspect simulation results in real time while running multiple simulations or to easily share and manage results. Here we present WebCM, a web-based platform for creating, editing, running, sharing, and interactively inspecting CellModeller simulations on a remote server via a web browser. Since it is browser-based, it enables users to access these features on many devices, including tablets and smartphones.

## Results

### Overview

WebCM is split into two parts: the user interface (front end) and the server (back end).

#### Front End

**Figure 1:**
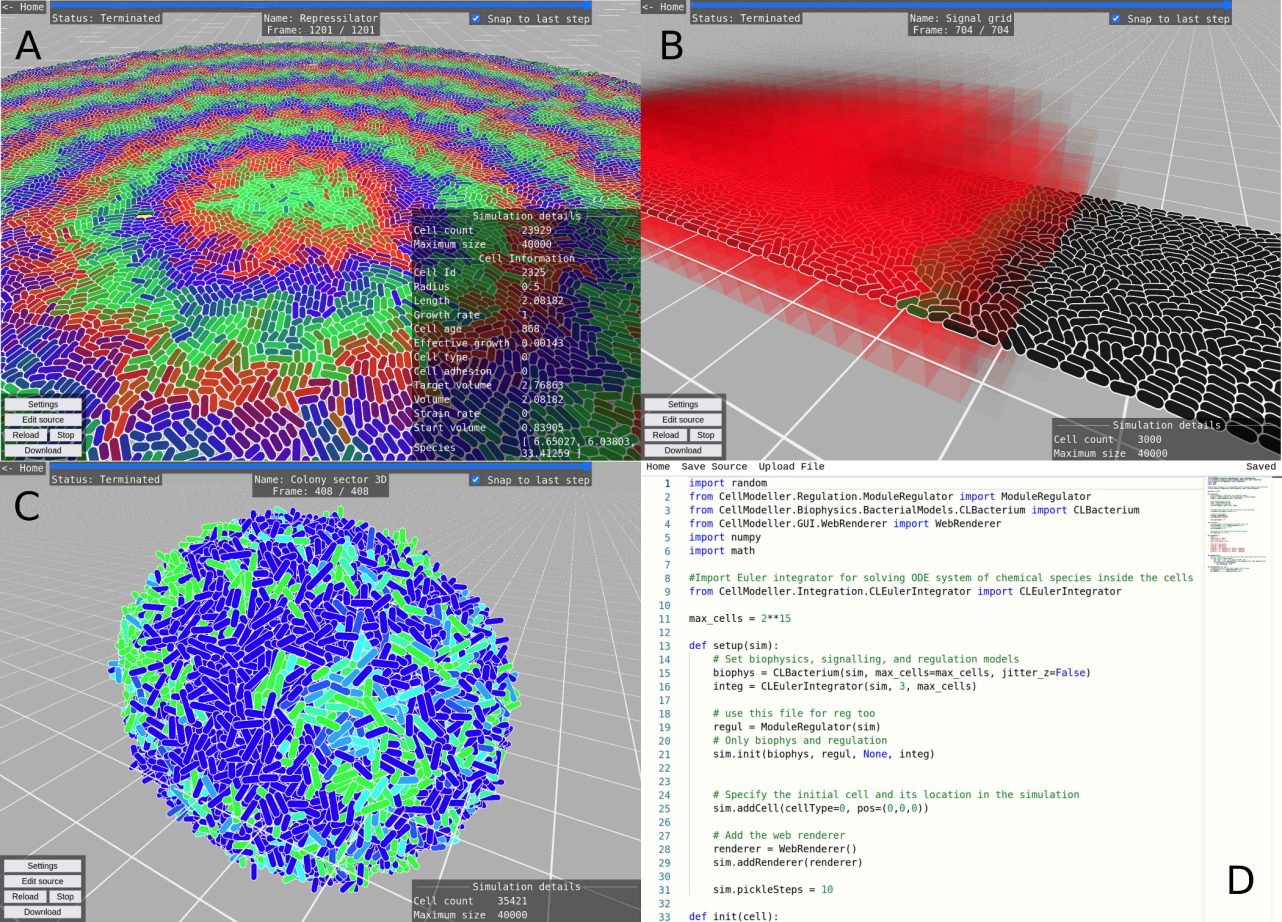
WebCM user interface. (A) Simulation of a surface-growing bacterial colony with the repressilator genetic oscillator circuit. Repressor protein concentrations are colored red, green, and blue. A cell is selected, and its properties are displayed to the right. The playback slider can be seen at the top. (B) Simulation of bacteria growing in a microfluidic channel and producing a diffusing signal (colored red). (C) Simulation of a three-dimensional bacterial colony. (D) Model editor page showing a CellModeller script.

The user interface (Figure 1) is divided into three sections:

1. Home page, which is a simple interface that allows users to create new simulations, view and share those that have been created, and delete existing ones.
2. Editor page, which is used to edit simulation scripts via a fully functional code editor (Figure 1D). Simulations can be saved and then run from the home page.
3. Visualization page, which allows users to view and interact in real time with their simulations in three dimensions, rotating, translating, and zooming as required. Selecting cells allows inspection of all cell properties.

A publicly accessible demonstration instance of WebCM is available at http://webcm.rudge-lab.org:8001. Users can log in as “guest” with the password “demo1234”. Local instances can easily be set up by following the instructions at github.com/RudgeLab/WebCM/blob/master/Documentation/INSTALL.md.

#### Back-End Server

**Figure 2:**
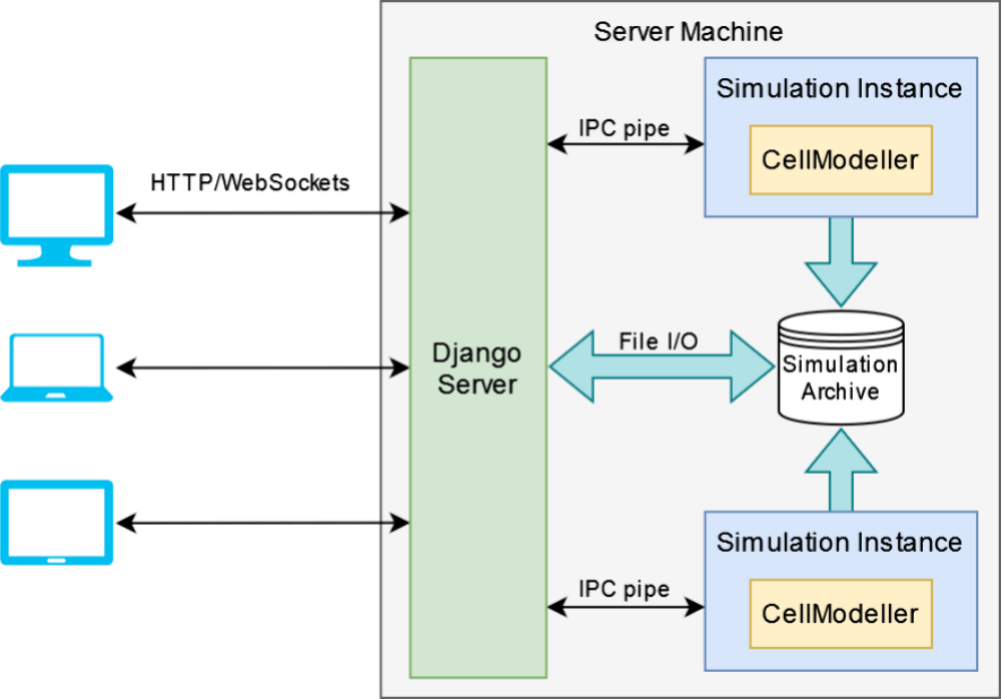
WebCM architecture. Clients (web browsers running on computers, tablets, or smartphones) communicate with a Django server by sending HTTP requests. The server creates simulation instances as separate processes with which it communicates via IPC pipes. Simulation results are saved at each step on the file system, where they can be retrieved by the server and sent to the client when requested. The server informs the client of new results (frames) using messages sent via WebSockets.

The server (Figure 2) is the remote computer responsible for running simulations and storing their results. It uses the standard CellModeller Python framework to run simulation scripts specified by the user via a web browser (client). The server sends the simulation results to clients, even after simulations have ended. It is able to run multiple simulations at the same time for each user and can retrieve their results as they run on request from the client.

### Architecture and Implementation

The architecture of WebCM is shown in Figure 2. Most of the communication between clients and the server is achieved through HTTP requests. This includes creating new simulations, listing all existing simulations, and pulling simulation results. The server also uses WebSockets to communicate with clients. This is done so that the server can send update notifications to the client, such as when new simulations results are available or when an error has occurred.

#### Running Simulations

When a new simulation is created, the server spawns a new process (Figure 2), which enables multiple simulations to be run in parallel. This process is known as a *simulation instance*. It is responsible for communicating with the server, stepping through the simulation, and saving the results of the simulation. Because the simulation instance is a separate process from the server, all communication has to be done through an interprocess communication (IPC) pipe. An IPC pipe is a simple mechanism that allows different processes to send data to each other.

A simulation instance is a simple wrapper around the CellModeller simulation software and is responsible for communicating with the server and saving simulation data. Each instance starts by initializing the simulation based on the parameters provided by the user in the model script and then begins stepping through the simulation. After each step, the instance saves the current state of the simulation and informs the server that a new frame has been generated. Each simulation instance runs as a separate process. This allows us to overcome the Python global interpreter lock (GIL), a mechanism that prevents multiple threads from executing simultaneously. This also means that simulations run independently from one another, so if one causes its process to crash, then the others can continue running.

CellModeller simulations are run in discrete steps. Each step updates the simulation in a small time span. After a step is completed, the results of the simulation can be read, analyzed, and saved. Then the next step is executed, and this process is repeated. In WebCM, each simulation step is referred to as a *frame*. When a new frame is produced, its contents are saved to a file, and an update notification is sent to the clients. Because simulation instances are in processes different from the server, this has to happen in two steps. First, the notification is encoded and sent over an IPC pipe to the server. Then, when the notification is received, the server processes it and broadcasts a corresponding message to all clients connected to the simulation. This notification is not sent to clients that are not connected to the simulation. All other messages between the simulation instance and the server are handled in a very similar manner.

#### Storing Simulation Results

Originally, CellModeller used a file format called a pickle. Pickle is a custom file format that is used by Python to store Python objects. It is a very versatile format, even allowing the storage of arbitrary Python code within objects that can be loaded and executed by another application. However, this versatility comes at a cost. Writing and reading data that are in the pickle format can take a long time and can take up a lot more space than required. The format is also not secure since it allows for arbitrary code execution. For these reasons, we decided to use *msgpack*, a binary alternative to JavaScipt Object Notation (JSON), for storing the simulation state. It is faster and takes up less space while providing the same level of flexibility.

Like JSON, msgpack stores the attributes of each object as key–value pairs. The keys are typically strings, which means that if there are many objects with the same attributes, there is a lot of duplication of data. To mitigate this issue, WebCM’s file exporter first collects all separate attribute names in a dictionary and assigns them indices. Duplicated keys are placed in the dictionary only once and are assigned the same index. These indices are used as the keys instead of the attribute names. When a file is imported, the dictionary is used to reverse the process and restore the original attribute names for each object.

#### Visualizing Simulations

WebCM also stores the simulation state in a second format, which is designed specifically for visualizing the simulation. Because of this, the format can be a lot more compact, requiring only the attributes of the simulation needed for visual rendering. It stores the position, direction, radius, length, color, and ID of each cell. The format is designed to require almost no processing by the client and, due to its smaller size, takes up less network bandwidth. These data are sent to the client via HTTP requests, enabling efficient visualization of simulations in real time. The front end then renders images of the simulation state at a given time and allows live user interaction such as moving the camera/viewpoint around and clicking on cells to view their attributes.

#### Integration of Other Computational Modeling Tools

Any simulation tool could be incorporated into the framework if it provides the required Python functions to produce descriptions of the cell states in the correct format. This means that different back-end simulation tools could be used by developing simple wrapper and data format conversion functions.

## Discussion

We have developed and implemented a system to effectively put CellModeller on the cloud in a way that enables multiple users to run multiple simulations in parallel and share, visualize, and edit them in real time. Users can run large simulations via a web browser even on minimal hardware, including tablets and smartphones, and do not have to install CellModeller on their own device (this is installed on the remote server). This will significantly increase access to advanced modeling tools for researchers studying synthetic and natural microbial communities, including those with limited programming and command line knowledge. It will democratize such tools by allowing multiple users to share the same hardware, reducing lab costs, and boosting collaboration and productivity. We note that the use of web applications to increase the accessibility of synthetic biology software is gaining popularity. The possibility of connecting such tools via their APIs will open up the possibility of complete workflows without the need for users to install software.

Because of the way that WebCM was designed, it is possible for it to host tools other than CellModeller. As long as the simulation tool produces compatible output, i.e., a list of individual cells and their properties, WebCM would allow visualization and inspection of the simulation in the same way. The interface between WebCM and any simulation tool is very small, so no major changes to the code base would be required. A simple wrapper for other simulation frameworks could be created that converts the results to the required format.

## Methods

The WebCM server was written using the Django Python web framework.^12^ All of the communication between the clients and the server is done via HTTP requests and WebSockets using the Django channels library. When deploying the server, uvicorn is the recommended framework; however, WebCM also supports running with Django’s Daphne server. The server uses the msgpack^13^ and numpy^14^ Python packages. The front-end client uses hypertext markup language (HTML) and JavaScript, and all the rendering is done using WebGL.^15^ We also use Microsoft Monaco web editor^16^ for editing simulation scripts in the browser.

## Data Availability

All code, documentation, installation instructions, and user guide can be found at https://github.com/RudgeLab/WebCM.

## Supporting Information Available

Movie showing an example simulation (MP4)
